# Antimicrobial stewardship interventions reduce the time to the first antibiotic administration in septic patients in ICUs: regional multicenter study in 7 Latin American high-complexity hospitals

**DOI:** 10.1128/aac.01850-24

**Published:** 2025-06-17

**Authors:** Juan Carlos García-Betancur, Christian José Pallares, Natalia Restrepo-Arbeláez, Elsa De La Cadena, Wanda Cornistein, Paula Susana Byró, Diogo Boldim-Ferreira, Rodrigo Ahumada, Nicolás Valdebenito, Jorge Chaverri-Murillo, Paulo Castañeda-Méndez, Itaivet Toledo, Elsa Yasmín Vente, Luis Hercilla, Vanessa Moreno, Debra A. Goff, María Virginia Villegas

**Affiliations:** 1 Grupo de Investigaciones en Resistencia Antimicrobiana y Epidemiología Hospitalaria (RAEH), Universidad El Bosque 28009, Bogotá, Colombia; 2 Clínica Imbanaco grupo Quirónsalud 173050 , Cali, Colombia; 3 Hospital Universitario Austral 218809, Buenos Aires, Argentina; 4 Hospital Universitario, Universidade Federal de São Paulo 28105, São Paulo, Brazil; 5 Hospital Dr. Gustavo Fricke 37617, Viña del Mar, Valparaiso, Chile; 6 Hospital Calderón Guardia 189692 , San José, Costa Rica; 7 Hospital Médica Sur 538694 , Mexico City, Mexico; 8 Hospital Nacional Alberto Sabogal Sologuren 280929 , Lima, Peru; 9 College of Pharmacy, The Ohio State University 15480 , Columbus, Ohio, USA; University of Pittsburgh School of Medicine, Pittsburgh, Pennsylvania, USA

**Keywords:** antimicrobial stewardship (AMS), antibiotics, administration times, hang time, Latin America, time-to-antibiotics, antimicrobial stewardship programs (ASP), intensive care units (ICU)

## Abstract

Delay in the initial administration of antimicrobials is one of the strongest predictors for mortality for septic patients in the intensive care unit (ICU). Given the different logistics among hospitals for antibiotic administration, this delay can take hours. As antibiotic administration involves care coordination and the participation of different team members, education and the antimicrobial stewardship (AMS) program play a key role in reducing these times. This study evaluated the time between the initial prescription and the effective administration of antibiotics for ICU patients in seven Latin American hospitals, before (pre-) and after (post-I and post-II) the implementation of a tailored educational approach. After establishing a baseline measurement (pre-), we implemented a tailored educational intervention directed to the ICU team including nurses, specialists, pharmacists, and the members of the AMS team. Then, we conducted a post-intervention measurement after a 3 month period (post-I) and repeated it after a 1 year period (post-II). During the pre-interventional phase, the hang time varied between 88 and 178 min, reporting an adherence to the 1 hour bundle of 33.8%. For the post-I, it significantly reduced with time variations between 46 and 104 min, showing an increase of 54.9% in adherence. After 1 year, in post-II, a persistent effect of shorter administration time was observed, varying between 49 and 109 min, increasing the adherence to 59.6%. Our results highlight that an active and tailored multidisciplinary AMS educational process incorporating antibiotic hang time protocols and including multidisciplinary healthcare teams involved in coordinating sepsis care decreases the administration time of antibiotics in Latin American hospitals with limited resources.

## INTRODUCTION

Severe sepsis and septic shock remain the main cause of mortality and morbidity in a variety of patients, independent of their socio-economic status, age, or geographical origin (1–4). Although there are different measurements for the “early appropriate antibiotic treatment” (5), the general rule is that delay of appropriate and timely antibiotic treatment is associated with higher mortality in critically ill patients in the intensive care unit (ICU) (3, 6–13). Among the most effective strategies to tackle this problem is the concept of a “golden hour,” which is the administration of the first dose of antibiotics within 1 hour after the diagnosis of sepsis (5, 7, 14–18). Current international guidelines, including the Surviving Sepsis Campaign (19), recommend initiation of appropriate antimicrobial therapy within this “golden hour” after the recognition of sepsis (20, 21).

Antimicrobial stewardship (AMS) interventions aim to optimize the appropriate use of antimicrobials, tackling antimicrobial resistance through improving the appropriateness of antibiotic use, including the correct diagnosis, dose, timing, and duration of therapy (22). Among these AMS interventions, the reduction in “hang time” to accomplish with the 1 hour bundle has shown to bring clear benefits to reduce mortality and improve proper antibiotic use for critical septic patients in the ICU (3, 23, 24). Indeed, hang time (24) is often used, together with other indicators, to quantify the success of any AMS program (ASP) in optimizing the outcome of ICU patients (25–28). Messina et al. defined “hang time” as the time elapsed from the written antibiotic order on a paper or digital chart to actual intravenous administration (24). Coordinating care for critically ill patients in the ICU is particularly challenging, as it requires seamless collaboration among multiple healthcare specialists and team members to achieve optimal clinical outcomes (29). Unfortunately, it has been shown that hang times can extend to several hours due to the complexities of sepsis care, including failures in hospital logistics, lack of communication between medical, nursing, and pharmacy, and the pharmacy delivery process. Luckily, most of the factors affecting long hang time can be approached and improved by establishing educational training, mentoring, feedback, and a conscious follow-up (24, 30–34).

In the Latin American (LATAM) context, the impact of AMS interventions has been well documented (35), including the initiative to create a Latin America network for clinical training and support of pharmacists involved in ASP hospitals (LASCONET) in different hospitals along LATAM countries (36, 37).

Within this regional initiative, the objective of this study was to measure the change in antibiotic hang time compliance with the “golden hour” bundle for antibiotic administration in septic patients in seven hospitals from seven different LATAM countries, before (pre) and after (post-I and post-II) the implementation of a multidisciplinary educational program for pharmacists, nurses, and the medical staff (37). The primary outcome was the reduction in hang time for the administration of the first antibiotic for septic patients in the ICU of these seven LATAM hospitals, measuring 3 months (post-I) and 1 year (post-II) post-intervention to determine if the AMS intervention was sustainable over time.

## MATERIALS AND METHODS

Our prospective study utilized a participatory research (38), interventional, multicenter, (quasi) experimental methodology to compare the time between the initial prescription and the time of the effective administration of antibiotics (which was defined for the study as the “hang time”) in patients with sepsis or septic shock diagnosis; a before and after tailored educational AMS intervention implementation was addressed to infectiology, nursing, and pharmacy teams working in the general adult ICU (medical, trauma, and surgical) of each participating hospital.

### Population

Inclusion criteria included adult patients admitted to the ICU who received a first dose of antibiotic, with suspected sepsis or septic shock, following the definition in current sepsis guidelines (19, 20, 39) secondary to urinary, intra-abdominal, soft tissue, or pneumonia infection. The study included seven LATAM high-complexity hospitals from Argentina (size facility 200 operational and 15 ICU beds), Brazil (size facility 400 operational and 110 ICU beds), Chile (size facility 560 operational and 42 ICU beds), Colombia (size facility 254 operational and 64 ICU beds), Costa Rica (size facility 512 operational and 33 ICU beds), Mexico (size facility 206 operational and 34 ICU beds), and Peru (size facility 672 operational and 16 ICU beds). At each of the seven participating hospitals, septic patients were identified by chart review and by the physician diagnosis based on clinical evidence such as hemodynamic compromise, systemic inflammatory response syndrome, organ dysfunction like oliguria, altered level of consciousness, hypotension (systolic blood pressure [SBP] <90 mmHg or mean arterial pressure [MAP] <65 mmHg), hypoxemia (recent or increased need for O2), complemented with any documented infection with either positive cultures, molecular tests, or any imaging study compatible with infection. Sepsis alerts and surveillance systems were not present in all hospitals, except Mexico and Peru.

### Data sources

Measures of the hang times in three-period phases was performed as follows: (i) pre-intervention phase from February 2023 to May 2023, (ii) a first post-intervention phase, or “post-I” from August 2023 to October 2023 and, (iii) a second post-intervention phase 1 year later, or “post-II” from August 2024 to October 2024. Data were collected by a single investigator from electronic medical records, anonymized, and exported to a standardized data collection template using spreadsheet software. Data were collected in real-time or retrospectively to include the 24 hours of the day, the 7 days of the week (including weekends), and verified to assure that the time of prescription versus administration of the antibiotic was correct on the electronic medical record. Hang time (24) was defined in three time frames: <60, referring to a hang time from 0 to 60 min; 60–120, referring to a hang time from 1 to 2 hours, and finally, >120, referring to a hang time longer than 2 hours. Data collected included admission date, diagnosis, type of antibiotic used, date and time of the written antibiotic order (defined as “time zero”), date and time of the dispensation of the antibiotic by the pharmacy, and date and time of the effective administration of the first dose of the antibiotic in the ICU. Data were collected during the pre-intervention phase (number of observations [*n*] ≥ 120 per ICU per hospital), post-I (*n* ≥ 120), and post-II (*n* ≥ 30). Hang time was measured as outcomes in these ICUs, and the average time difference between the pre-intervention and post-I and post-II phases was included in a statistical analysis for each hospital and to perform descriptive comparisons among hospitals.

### Intervention

Using a participatory research framework (38), the intervention was created by engaging and including healthcare providers for sepsis care in the seven hospitals. We required hospitals to have an infectious disease physician (ID) and a pharmacist as part of the AMS team to participate in the study. All the ASP members from each hospital also got involved, as they played an important role in the post-I and post-II educational process. A series of online meetings was carried out between our AMS experts and the ASP members from each participating institution. The tailored AMS educational strategy was implemented during the pre- and post-I phases and included online meetings during the pre-intervention focusing on (i) detailed description of the objectives and methodology of the study, (ii) how to record the observations during the pre-interventional and post-interventional phases, (iii) presentation and discussion of the results of the pre-interventional phase with the ICU healthcare professionals, emphasizing in the main problems and barriers found influencing the hang time and how to decrease the gap proposed by their own ICU team. The (iv) phase involved the recording of the hang time during the post-I phase and, finally, (v) a follow-up 1 year later during the post-II phase. During the post-I phase, a one-to-one educational online meeting took place between our two AMS experts (specifically, Dr. Goff and Dr. Villegas) and the complete AMS team from each participating hospital; the discussion was centered on how the particular problems and barriers found during the pre-interventional phase could be overcome and, in case the barriers persisted, how they would approach them. The follow-up of the hang time data observations was done 3 months (post-I) after the educational intervention in the post-I phase, as well as a 1 year evaluation of the hang time observations (post-II). None of the post-I and post-II had further educational intervention.

### Statistical analysis

All data records were collected, tabulated, and reviewed by the co-authors. To compare pre-intervention and post-I and post-II hang time data, statistical analysis was performed using Jamovi version 2.3.21.0 software (40) and the non-parametric Mann-Whitney U-test to determine significance in overall hang times. *P* < 0.05 was considered statistically significant.

## RESULTS

We measured 3,793 antibiotic orders in the seven LATAM hospitals in a period of 20 months (from February 2023 to October 2024) divided into three different phases, as described in Materials and Methods. In general terms, all seven LATAM hospitals showed a significant reduction (*P* < 0.05) in their hang times to accomplish the 1 hour bundle.

During the pre-interventional phase (with 877 observations), hang time was 128 min on average, with the majority (66.2%) of the hang time observations exceeding 60 min, as seen in Table 1. Only 33.8% of the observations in the seven hospitals fell in the <60 time frame, 28% of the observations ranged in the 60–120 time frame, and 38.2% in the >120 time frame. During the pre-interventional phase, the time range for all seven countries was between 1 min and 1,453 minutes. Chile showed the longest average time among all LATAM countries (178 min), while Argentina showed the shortest average time to the first administration, with 88 min. In the case of Mexico, 55.6% of the administrations fell in the <60 time frame, while Peru showed the lowest number of observations <60 time frame, with only 20%. For the time frame that exceeded more than 2 hours (>120 time frame), 60% of the observations belonged to the Brazilian hospital, in contrast to only 14.8% of the Argentinian hospital (Table 1).

**TABLE 1:** Hang time for seven LATAM hospitals during the pre-intervention phase^*a*^

	Pre-intervention									
		Time^*b*^						Average time (min)	Median (min)	Range (min)
	*N* =	<60 min		60–120 min		> 120 min				
		*n*	%	*n*	%	*n*	%			
Argentina	162	52	32.1	86	53.1	24	14.8	88	78	19–279
Brazil	120	32	26.7	16	13.3	72	60.0	173	144	2–620
Chile	156	46	29.5	27	17.3	83	53.2	178	133	2–1,086
Colombia	120	41	34.2	34	28.3	45	37.5	123	91	2–540
Costa Rica	57	22	38.6	18	31.6	17	29.8	108	74	23–510
Mexico	142	79	55.6	32	22.5	31	21.8	89	50	1–1,453
Peru	120	24	20.0	36	30.0	60	50.0	138	119	23–480

^*a*^ Time ranges were defined between 0 and 60 min (<60 min), 60 to 120 minutes (60–120 min), and longer than 120 min (>120 min), average hang time, median, and time range.

^*b*^ Defined as the time between prescription and administration of the selected antimicrobials in the ICU.

After the pre-interventional phase, following the tailored educational strategies, 825 observations were recorded during the post-interventional phase or post-I (August 2023), as seen in Table 2. In this post-I, the average net reduction for all seven hospitals was 78 min (from 128 to 53 min), confirming the benefit of these tailored AMS educational interventions. In fact, there was a statistically significant reduction in the average hang time for six out of the seven participating hospitals. The Argentian hospital showed the lowest average time to the first administration, with 46 min, being 77.9% of its hang time <60 time frame. Despite the fact that Peru showed the longest average time among all seven LATAM hospitals during the post-I phase (from 138 to 104 min), there was a significant decrease (*P* = 0.05) in their hang time. Despite the important decrease in the length of the time range for all seven hospitals, a broad time frame was still seen, from 1 to 1,300 min (Table 2). During post-I, 54.9% of all administrations for all seven hospitals fell in 1 hour (<60 time frame), 25.8% between 1 and 2 hours, and only 19.3% over 2 hours.

**TABLE 2:** Hang time for seven LATAM hospitals during the first post-intervention phase, post-I (3 months after the AMS intervention)^*a*^^,^^*c*^

	Post-intervention I										Net reduction (from average)		*P*-value	
		Time^*b*^						Average time (min)	Median (min)	Range (min)				
	*N* =	<60 min		60–90 min		>120 min								
		*n*	%	*n*	%	*n*	%				(min)	(%)		
Argentina	131	102	77.9	23	17.6	6	4.6	46	40	10–150	42	48	<0.001	***
Brazil	120	57	47.5	33	27.5	30	25.0	87	60	0–680	86	50	<0.001	***
Chile	128	63	49.2	38	29.7	27	21.1	88	62	7–974	90	302	<0.001	***
Colombia	120	83	69.2	28	23.3	9	7.5	51	41	3–179	72	59	<0.001	***
Costa Rica	47	24	51.1	19	40.4	4	8.5	62	58	15–155	46	43	0.011	*
Mexico	159	95	59.7	32	20.1	32	20.1	85	40	1–1,300	4	4	0.651	n.s.^*d*^
Peru	120	36	30.0	26	21.7	58	48.3	104	77	27–435	34	25	0.005	**

^*a*^ Differences in average hang time for each participating hospital were measured as net reduction when compared to the pre-intervention phase (Table 1), and statistical significance was determined. *P* < 0.05 was considered significant.

^*b*^ Defined as the time between prescription and administration of the selected antimicrobials in the ICU.

^*c*^ * = *P* ≤ 0.05, ** = *P* ≤ 0.01, *** = *P* ≤ 0.001.

^*d*^ n.s indicates non statistically significant.

To validate that these AMS interventions persisted, we measured again the time to the first dose of antibiotics (with 389 observations) after 1 year, or “post-II” (August 2024), as seen in Table 3. During post-II, we found a net reduction of nearly 1 hour (59.9 min). Here, the pattern of statistically significant reduction (*P* < 0.05) persisted, when compared to the pre-interventional phase, suggesting that the AMS intervention was durable over time. Furthermore, the average hang time was even lower than during phase I (68 min on average) with ranges from 0 min to 426 (Table 3). In this post-II, 59.6% of the administrations fell in the time frame <60, with Colombia being the hospital with the highest percentage (80%) in this time frame; in contrast, Costa Rica had only 22.4% of the administrations below 1 hour, 25.8% between 1 and 2 hours (60–120 time frame), and only 14.6% of the measures in the >120 time frame.

**TABLE 3:** Hang time for seven LATAM hospitals during the second post-intervention phase, post-II (1 year after post-I)^*a*^

	Post-intervention II (after 1 year)										Net reduction (from average)		*P*-value	
		Time^*b*^						Average time (min)	Median (min)	Range (min)				
	*N* =	<60 min		60–120 min		>120 min								
		*n*	%	*n*	%	*n*	%				(min)	(%)		
Argentina	118	69	58.5	31	26.3	18	15.3	70	53	0–426	18	20	<0.001	***^c^
Brazil	30	20	66.7	6	20.0	4	13.3	64	60	0–163	109	63	<0.001	***
Chile	49	26	53.1	19	38.8	4	8.2	64	59	8–202	114	64	<0.001	***
Colombia	30	24	80.0	3	10.0	3	10.0	49	23	2–312	74	60	<0.001	***
Costa Rica	58	13	22.4	22	37.9	23	39.7	109	78	40–240	-1	-1	0.088	n.s.^*d*^
Mexico	75	48	64.0	15	20.0	12	16.0	67	45	0–275	22	25	0.575	n.s.
Peru	29	21	72.4	8	27.6	0	0.0	55	53	24–107	83	60	<0.001	***

^*a*^ Differences in average hang time for each participating hospital were measured as net reduction when compared to the pre-intervention phase (Table 1), and statistical significance was determined. *P* < 0.05 was considered significant.

^*b*^ Defined as time between prescription and administration of the selected antimicrobials in the ICU.

^*c*^ *** = *P* ≤ 0.001.

^*d*^ n.s. indicates non statistically significant.

## DISCUSSION

The present study is the first, to the best of our knowledge, to systematically measure hang times in LATAM countries and to evaluate the impact of tailored AMS educational interventions in reducing the administration of the first dose of antibiotics in septic patients hospitalized in the ICU. This study showed a significant reduction in antibiotic hang time, improvement which was sustained for 1 year across all seven participating hospitals in LATAM countries. We surveyed 3,793 records of septic patients who received the first dose of antibiotics, evaluating a pre-interventional phase and two post-interventional phases after an AMS-tailored educational intervention. A considerable delay in the administration of the first dose of antibiotics to ICU septic patients in all seven LATAM hospitals was seen in the pre-interventional phase, with a mean value of more than 2 hours. The importance of timing and appropriateness of the first dose of antibiotics in septic patients has been extensively shown (3, 8, 17, 41, 42). The Surviving Sepsis Campaign guidelines (19, 20) recommend the administration of effective intravenous antimicrobials within the first hour of septic shock or severe sepsis. Despite the fact that the seven hospitals are recognized as excellence centers for AMS in LATAM (37) and that all have AMS guidelines implemented, the “golden hour,” the “hang time,” was never measured for septic patients in the ICU. As observed in the results, and to the surprise of the ASP as well as the ICU team, hang time hours were particularly high, and not in the “golden hour” time frame for all seven hospitals in the LATAM countries.

During the AMS educational intervention, we found multiple barriers related to the delay in the timely administration of the first dose of antibiotics. Among the limitations found by the hospitals, the three most important ones were the lack of effective communication between the ICU physicians and the nurses to act upon a septic patient code, the limitation of the availability of the antibiotics needed, and the lack of awareness of the pharmacy to dispense on time the antibiotic for a septic patient. A detailed list of factors and barriers reported by the participating ASP teams is listed in Table 4. Once these limitations were identified, the education of the personnel involved in the prescription, order, dispensation, and administration of antibiotics in the ICU was shown to be an effective strategy to improve care coordination in septic patients and an effective way to decrease barriers contributing to the antibiotic delay, as shown in Table 1, as well as to confirm the success of the educational intervention, as shown in Tables 2 and 3 (43).

**TABLE 4:** List of main factors or barriers associated with delay in hang times across the seven LATAM hospitals reported by the participating ASP teams

List of main factors or barriers associated with delay in hang times across the seven LATAM hospitals	
Factor or barrier	LATAM hospital reporting
Pharmacists do not work directly within the ICU.	All seven hospitals
Drug delivery of antibiotics to the ICU.	All seven hospitals
Lack of collaboration and active communication between physicians, nurses, and pharmacists.	All seven hospitals
Time constraints.	All seven hospitals
Absenteeism, constant changes, and turnover of medical, pharmacy, and nursing professionals.	Brazil and Costa Rica
Lack of AMS training of new medical residents and ICU physicians.	Brazil and Colombia
Subjective clinical assessment of infection among individual patients.	Chile and Peru
Outdated computer systems.	Argentina
Pharmacy department with limited staff and technology.	Argentina
Lack of electronic records for antibiotic administration times.	Chile
Shortage of some antimicrobials.	Costa Rica

Advanced ASP interventions using an interprofessional mentoring program have shown to be effective in reducing hang times (32). Goff et al. emphasize the need for collaboration and engagement of the different healthcare professionals, including nursing and pharmacy team members, through face-to-face meetings with an experienced mentor as well as follow-up meetings. Following the success of the Goff et al. experience, we implemented several interventions by online meetings with our international ASP ID experts, the pharmacist, nursing personnel, the ICU team, and the ASP hospital team members, which included discussion of the results and a customized approach to overcome the limitations shown in the data obtained in the pre-intervention period. Kalich et al. also demonstrated that implementing an updated antibiotic-specific sepsis bundle, with antibiotics put in an automated medication cabinet, resulted in improvements in the initiation of appropriate initial antibiotic therapy for severe sepsis in the emergency department. In our study, we also addressed the need for the appropriate antibiotic within 1 hour of a septic patient in each of our selected hospitals (44). Due to time and resource limitations, we did not measure the association between the correct time for the initiation of antibiotic therapy and patient mortality. Further research may include this important outcome of patient mortality associated with hang time.

Our study considered two different endpoints (first endpoint, 3 months after the intervention [Table 2], and second endpoint, 1 year after the intervention [Table 3]) to measure the success of the educational intervention and the sustainability of the adherence to the hang time within the first dose of antibiotics in septic patients in the ICU. Despite the slight increase in hang times, from 53 min in post-I to 68 min in post-II 1 year later, compared to the pre-interventional phase, both were significantly lower and in compliance with the implementation of the strategies discussed and designed during the AMS educational intervention. The improvement of the hang times below 60 min in septic patients proved that the seven institutions took a proactive approach in their clinical practices for these critical patients.

Another important aspect not strictly approached here was the time to effective empiric antibiotic therapy (TTET), since our study was not intended to measure the current antibiotic administration guidelines from any of the participating hospitals nor to measure mortality as stated before. Nevertheless, it is important to mention that for suspected sepsis and septic shock, the right effective empiric antibiotic regimen should be given within the first hour. Another important factor for future research is the integration of rapid diagnostic point-of-care, such as molecular tests, to reduce hang times and increase TTET, for timely and appropriate empiric therapy. Timely clinical decision-making, based on available clinical and laboratory and point-of-care diagnosis information, patient history, and clinical guidelines, plays a critical role in selecting empiric therapy that is both effective and appropriate, helping to achieve the optimal TTET. Striking a balance between timely and tailored antimicrobial therapy is crucial to start effective empiric therapy promptly, the ability to de-escalate, and provide more targeted therapies to critical patients. Balancing these goals can minimize unnecessary broad-spectrum antibiotic use, thus supporting both clinical and stewardship objectives. The concept of TTET intersects with AMS and ASP goals, as timely therapy should still be aligned with minimizing the risk of resistance and preserving the effectiveness of antibiotics, particularly in ICU settings and for ICU patients.

The multidisciplinary work of prescription, ordering, and effective administration of the antibiotics within 1 hour plays a role in determining if initial antibiotic therapy is appropriate or not. Kalich et al. also showed that delays were greater in patients who were not seen by an emergency physician, those in whom the diagnosis of sepsis was not considered initially, and in those whose therapy was delayed while awaiting the performance of investigations (45). On the other hand, Hatozaki et al. did an educational intervention with the nursing staff, which included training sessions and feedback, to ensure early detection and management of patients with suspected sepsis, showing that it improved the early administration of antibiotics for patients with suspected sepsis (46). In our study, involving ICU pharmacists and nurses as part of the effective strategy to improve hang time was equally important for success. According to a recent self-assessment of AMS activities, implementation of strategies to promote multidisciplinary teamwork was uncommon in LATAM hospitals (47). Our study strategy of tailoring the AMS education intervention as teamwork showed positive results, as all hospitals showed a significant decrease in their hang times that lasted for a year. Although a success rate of 100% of antibiotic administration within 1 hour in the participating hospitals was not achieved within a year of the educational intervention, this study demonstrates that AMS strategies persist in time and tend to improve administration times, as factors and barriers, as those presented in Table 4 are iteratively corrected by the AMS teams. It also suggests that continuous healthcare education should be an ongoing strategy to keep the improvement shown.

The results obtained in the present study and the factors identified to influence the delay in the administration of the first dose of antibiotics to septic patients in the ICU are of fundamental importance to lead the improvement in the care of these critically ill patients in hospitals implementing ASP. Novel and tailored educational AMS strategies, as well as mentoring follow-up with feedback, must be considered as part of a teamwork strategy (48). Simple but effective actions from the ASP can make a huge impact in the quality of care of ICU patients, even decreasing mortality (19, 49–51).

Despite the fact that our study design did not measure the relationship between hang time and mortality, reduction in hang time is the first step.

This study faced limitations. Our study involved a group of seven different health institutions across LATAM, a geographical area characterized by a notable diversity in culture, hospital-assigned economic resources, and clinical practices. In addition to this variability, our seven hospitals included small private hospitals and big public university hospitals, making the variability in ICU practices, resources, and infrastructures important limitations to consider, which might have affected the implementation and success of our educational interventions. Differences in healthcare culture, physician-patient relationships, and attitudes toward antibiotic use may influence how the interventions were adopted and implemented. Also, differences in staff availability and turnover, accessibility to diagnostic tools, or hospital protocols could have influenced the baseline time to antibiotic administration and the extent of the improvement observed. Although the results obtained during the post-I and post-II phases were highly (and statistically) satisfactory, results cannot be extrapolated to all LATAM hospitals. We might consider a selection bias for this study, since all participating hospitals have been largely recognized as excellence centers for ASPs (belonging to the LASCONET network). Factors inherent to the ICU setting, such as patient severity, comorbidities, availability of point-of-care diagnostic tools, and clinical presentation, could have affected hang times. These confounders were not strictly controlled, and therefore may have affected the hang times results, making it difficult to compare among the different hospitals. Addressing these limitations through a more robust study design, control groups, long-term follow-up, and detailed statistical analysis could help strengthen the validity of the findings in future studies.

### Conclusions

Implementing multidisciplinary AMS interventions such as tailored education with follow-up and feedback to the ICU team taking care of septic patients significantly improved antibiotic hang times in ICUs. Further research is necessary to identify the impact of these interventions on outcomes, such as mortality. It is important to highlight that compliance with hang times less than 1 hour relies on the rapid diagnosis and treatment of sepsis, verification and dispensing of antibiotics by a pharmacist, and finally, administration of antibiotics by nursing staff. Utilizing existing resources and educational AMS interventions improved the administration of initial antibiotic therapy.
